# W/WO_3_/TiO_2_ Multilayer Film with Elevated Electrochromic and Capacitive Properties

**DOI:** 10.3390/ma18010161

**Published:** 2025-01-03

**Authors:** Zhenxing Wang, Guofeng Liu, Chonghui Li, Mei Qiao, Meng Tian, Xiaohui Lin, Wanling Cui, Xiaoxin Wang, Jinhai Liu, Shicai Xu

**Affiliations:** College of Physics and Electronic Information, Shandong Key Laboratory of Biophysics, Dezhou University, Dezhou 253023, China; gfliu26@163.com (G.L.); chonghuili163@163.com (C.L.); qiaomeirr@sina.com (M.Q.); tianmengdzu@163.com (M.T.); xiaohuilinsci@163.com (X.L.); wanlingcui@163.com (W.C.); wxx19871987@163.com (X.W.); liujinhai568@126.com (J.L.)

**Keywords:** electrochromic capacitive properties, multilayer structure, versatile color tunability

## Abstract

Electrochromic capacitors, which are capable of altering their appearances in line with their charged states, are drawing substantial attention from both academia and industry. Tungsten oxide is usually used as an electrochromic layer material for electrochromic devices, or as an active material for high-performance capacitor electrodes. Despite this, acceptable visual aesthetics in electrochromic capacitors have almost never been achieved using tungsten oxide, because, in its pure form, this compound only displays a onefold color modulation from transparent to blue. Herein, we have designed W/WO_3_/TiO_2_ multilayer films by a magnetron sputtering device. The impact of TiO_2_ layer on the optical and electrochemical properties was investigated. The results show that the optimum thickness of the TiO_2_ layer is 10 nm. The as-prepared film displays a high coloration efficiency (CE) of 74.2 cm^2^ C^−1^, a high areal capacitance of 32.0 mF/cm^2^, an excellent rate performance (with the areal capacitance still retaining 87% of the maximum capacitance at a current density of 1 mA/cm^2^), and a high cycle life (with a capacity retention of 91% after 1000 cycles).

## 1. Introduction

Electrochemical capacitors are among the key energy storage technologies that significantly contribute to addressing the pressing need for high-power-density solutions [1,2]. Supercapacitors can be divided into double electric-layer capacitors (DELCs) and pseudocapacitors [3,4]. Wang et al. reported activated carbon fibers (ACFs) with high micropore specific surface area and micropore volume that showed a high specific capacitance of 249 F g^−1^ at a current density of 0.05 A g^−1^ [5]. Thillaikkarasi, D. et al. fabricated an electrical double-layer capacitor with biomass-derived activated carbon (AC) and multi-walled carbon nanotubes (MWCNTs) that showed outstanding performance [6]. Pseudocapacitors achieve energy storage through reversible redox reactions [7,8]. Compared with double electric-layer capacitors that rely solely on the adsorption/desorption of ions to store energy, pseudocapacitors have higher specific capacity and broader application prospects. Pham, H.D. et al. designed a potassium-ion capacitor (KIC) using layered potassium niobate (K_4_Nb_6_O_17_, KNO) nanosheet arrays as anode and orange peel-derived activated carbons (OPACs) as fast capacitive cathode materials delivering both a high energy density of 116 Wh/kg and a high power density of 10,808 W/kg [9]. Some pseudocapacitive electrode materials can undergo reversible changes in ionic valence during charging and discharging processes, which can be accompanied by the generation of electrochromic (EC) phenomena [10,11]. Similar to the working principle of pseudocapacitive electrode materials, this type of electrochromic phenomenon is closely related to the intercalation and deintercalation of ions/electrons in active materials under external electric fields [12].

Supercapacitors with electrochromic properties can display the energy storage state of the supercapacitor through color changes [12,13,14,15,16]. For both supercapacitors and electrochromic devices, electrode materials, as the core of the device, directly affect the performance of the device. Transition metal compounds, characterized by high theoretical specific capacitance, rich valence states, easily obtainable raw materials, and low preparation costs, are often used as electrode materials for electrochromic supercapacitors. WO_3_ is frequently utilized in electrochromic supercapacitors, as it functions effectively as an active material for both establishing benchmarks in electrochromic device performance and enhancing the efficiency of supercapacitor electrodes [17]. However, the low conductivity and easy agglomeration of transition metal compounds generally limit their electrochemical performance.

The short service life limits the application of WO_3_ films. The electrochemical activity of WO_3_ film decreases with the process of charging and discharging [18]. The high stability and biocompatibility of TiO_2_ have attracted the attention of researchers. It has been reported that the service life of WO_3_ films can be improved by doping TiO_2_ [18,19,20,21]. However, the concentration of Ti doping seriously affects the properties of WO_3_ films [22].

Herein, W/WO_3_/TiO_2_ electrochromic supercapacitor complex films were fabricated by PVD, and the properties of these films were examined. The metal layer causes a distinct interference resonance, thus displaying a distinct structural color. The surface TiO_2_ layer can effectively prevent the WO_3_ layer from corrosion by acidic electrolytes. The interface effect of the hierarchical structure of W/WO_3_/TiO_2_ film provides a convenient method for the diffusion and charge transfer of ions. Complex films of W/WO_3_/TiO_2_ are capable of altering their structure in response to charge/discharge cycles, while also exhibiting superior electrochemical and electrochromic properties. These properties include elevated coloration efficiency, extended cycle life, and enhanced areal capacitance, all of which are achieved with the optimal thickness of the TiO_2_ layer.

## 2. Materials and Methods

ITO glass substrates (South China Science & Technology Co., Ltd., Shenzhen, China) were cut to a size of 2.0 × 1.0 cm^2^ and were used as electrodes. Before use, the ITO glass substrates were ultrasonically cleaned in acetone solution for 15 mins, in anhydrous alcohol for 15 min, and then in deionized water for 15 min. Finally, the substrates were dried in a nitrogen atmosphere. The W/WO_3_/TiO_2_ (10 nm) multilayer film electrodes were fabricated by a magnetron sputtering device. The details of the synthetic procedure were as follows: Metallic tungsten (W) films were deposited on clean ITO glass substrates by sputtering the W target (99.9%, ZhongNuo Advanced Material (Beijing) Technology Co., Ltd., Beijing, China) at 100 W and under 0.2 Pa. All WO_3_ films were prepared on W film by sputtering the W target (99.9%) at 100 W under 0.3 Pa. Then, TiO_2_ films were prepared on WO_3_ film by sputtering the TiO_2_ target (99.99%, ZhongNuo Advanced Material (Beijing) Technology Co., Ltd., Beijing, China) at 100 W under 0.3 Pa. By adjusting the deposition duration, the TiO_2_ layer was manipulated to range between 0 nm and 40 nm. The thickness of the TiO_2_ film was set at 0 nm, 6 nm, 10 nm, 20 nm, 30 nm, and 40 nm, corresponding to samples labeled as Film 1, Film 2, Film 3, Film 4, Film 5, and Film 6, respectively. The thickness and growth rate of the films were monitored using a quartz crystal oscillator film-thickness apparatus (FTM-V, Taiyao Vacuum Tech, Shanghai, China).

A field emission scanning electron microscope (FESEM, ZEISS MERLIN Compact, Jena, Germany) was used to observe the morphology of the films. The crystal and phase formations were recorded by X-ray diffraction (XRD, Bruker D8 ADVANC, Karlsruhe, Germany) with Cu Ka radiation (λ = 0.15405 nm), and the scanning speed was 2°/min. The optical properties of the films were recorded by a fiber optical spectrometer (AvaSpec-Mini2048CL-V125, Avantes, Beijing, China). The three-electrode system of an electrochemical workstation (CS310; Wuhan Corrtest Instruments Corp, Ltd., Wuhan, China) was employed in a 1 M H_2_SO_4_ solution for the electrochromic and electrochemical performance measurements of the hierarchical structure films. During these measurements, platinum (Pt) and silver/silver chloride (Ag/AgCl) were utilized as the counterelectrode and reference electrode, respectively. All optical images of complex film were obtained by camera.

## 3. Results and Discussion

**Figure 1:**
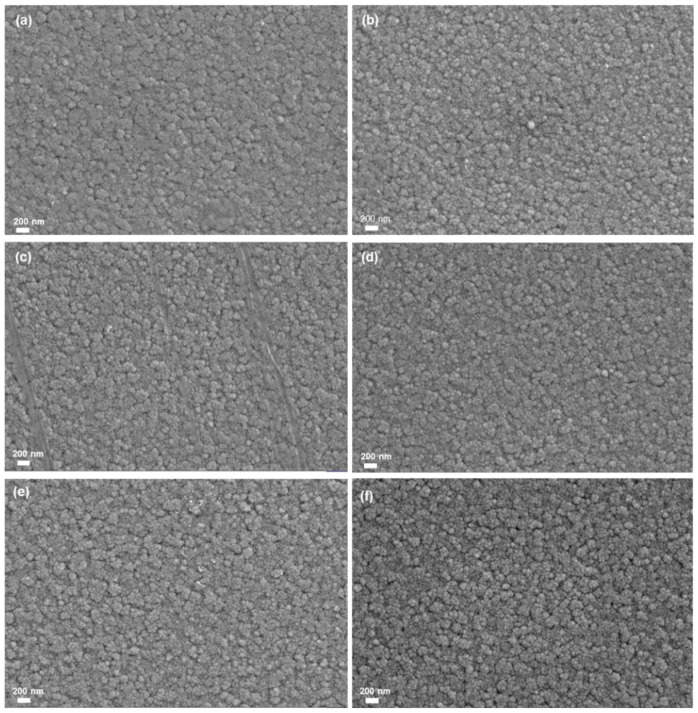
SEM micrographs of different samples: (**a**) Film 1, (**b**) Film 2, (**c**) Film 3, (**d**) Film 4, (**e**) Film 5, (**f**) Film 6.

**Figure 2:**
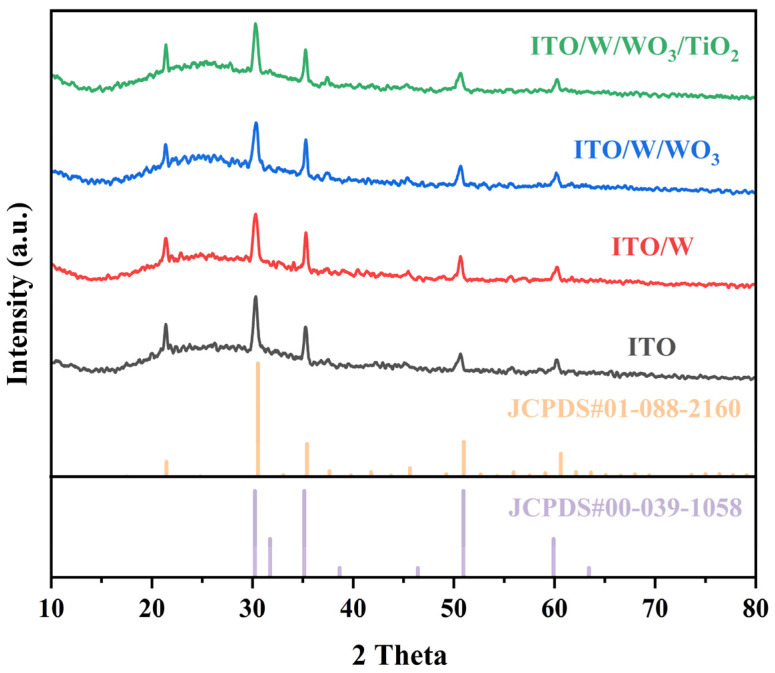
XRD patterns of ITO, ITO/W film, ITO/W/WO_3_ film, and ITO/W/WO_3_/TiO_2_ hierarchical structure film.

As can be seen from Figure 1, all films have a smaller nanoparticle size and exhibit uniform, compact, and smooth features. However, there are some differences in the morphology of different samples. As can be seen from Figure 1, the surface of the ITO/W/WO_3_/TiO_2_ complex films are rougher than that of the ITO/W/WO_3_ film, and the surface of the sample becomes rougher as the thickness of the TiO_2_ increases. The phase composition of individual ITO substrates, ITO/W, ITO/W/WO_3_, and ITO/W/WO_3_/TiO_2_ complex film, was characterized using X-ray diffraction (XRD), as shown in Figure 2. The XRD patterns of all films are highly consistent with the ITO (JCPDS#01-008-2160 and JCPDS#00-039-1058). The XRD patterns reveals no distinct characteristic peaks of the WO_3_ or TiO_2_ layers, indicating the amorphous structure of the WO_3_ and TiO_2_ layers.

**Figure 3:**
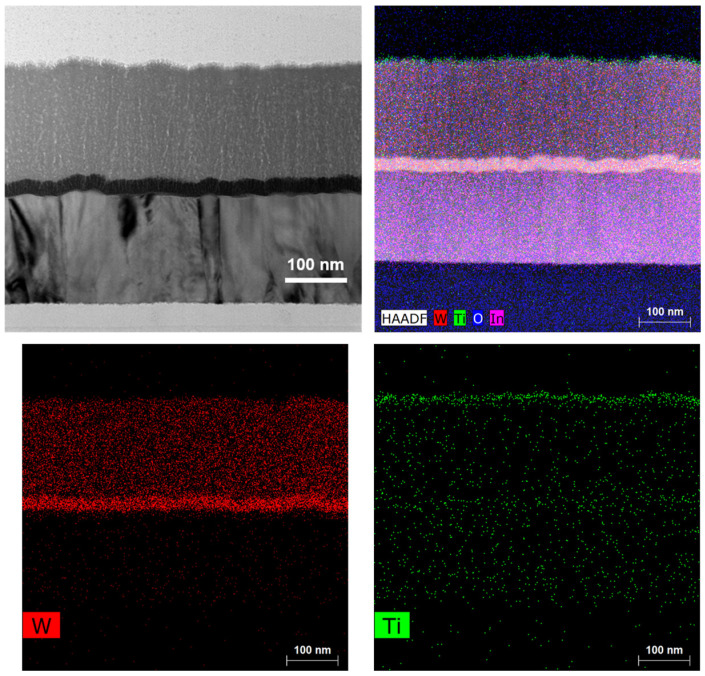
Structural characterizations of ITO/W/WO_3_/TiO_2_: cross-sectional SEM and corresponding elemental mapping images of W, O, and Ti.

The cross-section SEM image of the electrode in Figure 3 shows a perfect three-layer structure consisting of a thin W layer, a thin WO_3_ layer, and a thin TiO_2_ layer, with small thickness variation. The corresponding EDX mapping also indicates that a uniform distribution is achieved for the thin W layer, the thin WO_3_ layer, and the thin TiO_2_ layer in the ITO/W/WO_3_/TiO_2_ film. The thickness of the film, measured by a quartz crystal oscillator film-thickness apparatus, is consistent with that measured by the cross-section SEM image.

**Figure 4:**
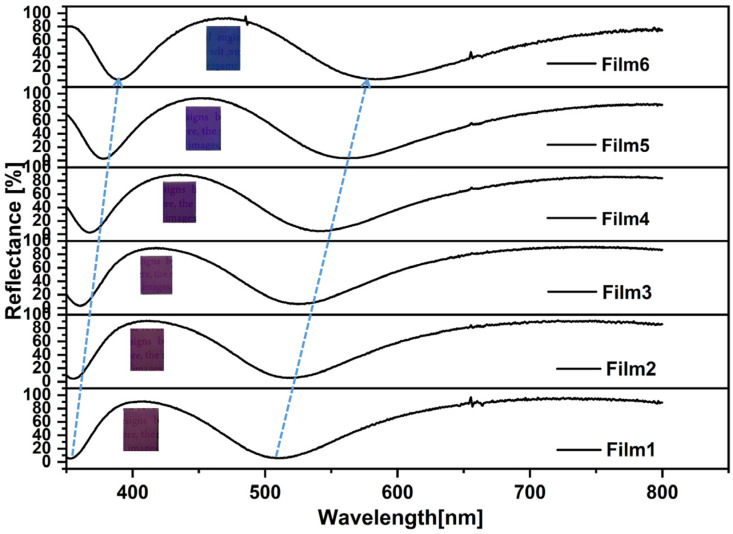
Reflection spectra (inset: optical images) of different samples: Film 1, Film 2, Film 3, Film 4, Film 5, Film 6.

Figure 4 shows the reflection spectra of Film 1, Film 2, Film 3, Film 4, Film 5, and Film 6. For the electrode with a 0 nm TiO_2_ thickness, there are multiple reflectance peaks and valleys in the reflection spectrum across the wavelength range of 350–800 nm, which are caused by the tungsten metal layer. Upon increasing the thickness of the TiO_2_ layer, the location of the reflection peaks and valleys are redshifted. The color changes from violet to blue by increasing the thickness of the TiO_2_.

**Figure 5:**
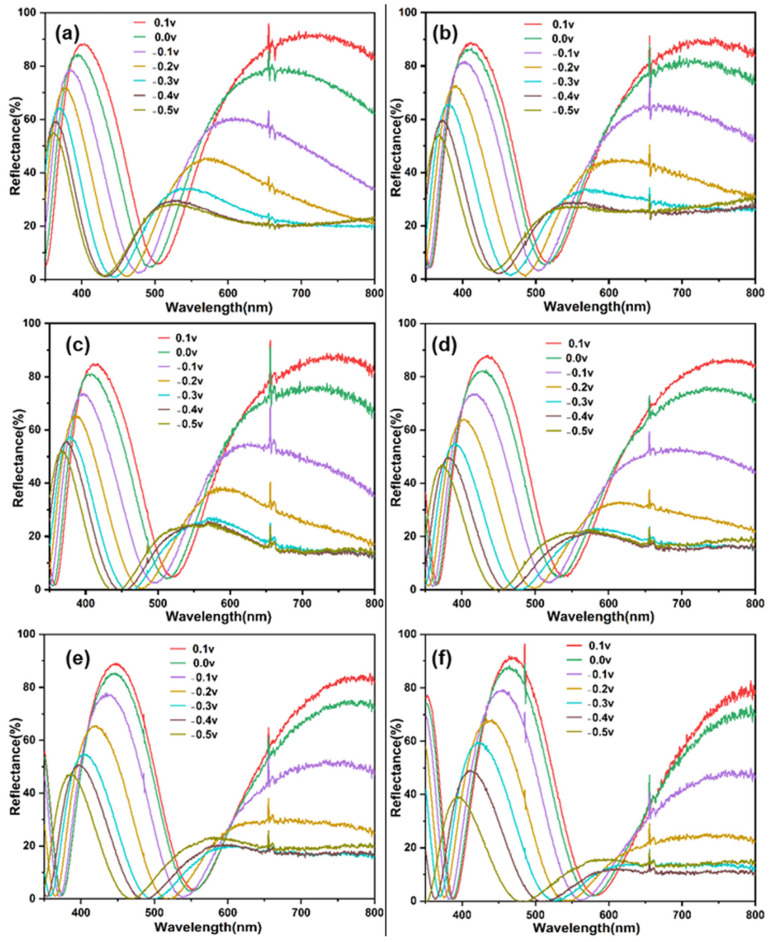
Reflection spectra of different samples with different potentials: (**a**) Film 1, (**b**) Film 2, (**c**) Film 3, (**d**) Film 4, (**e**) Film 5, (**f**) Film 6.

**Figure 6:**
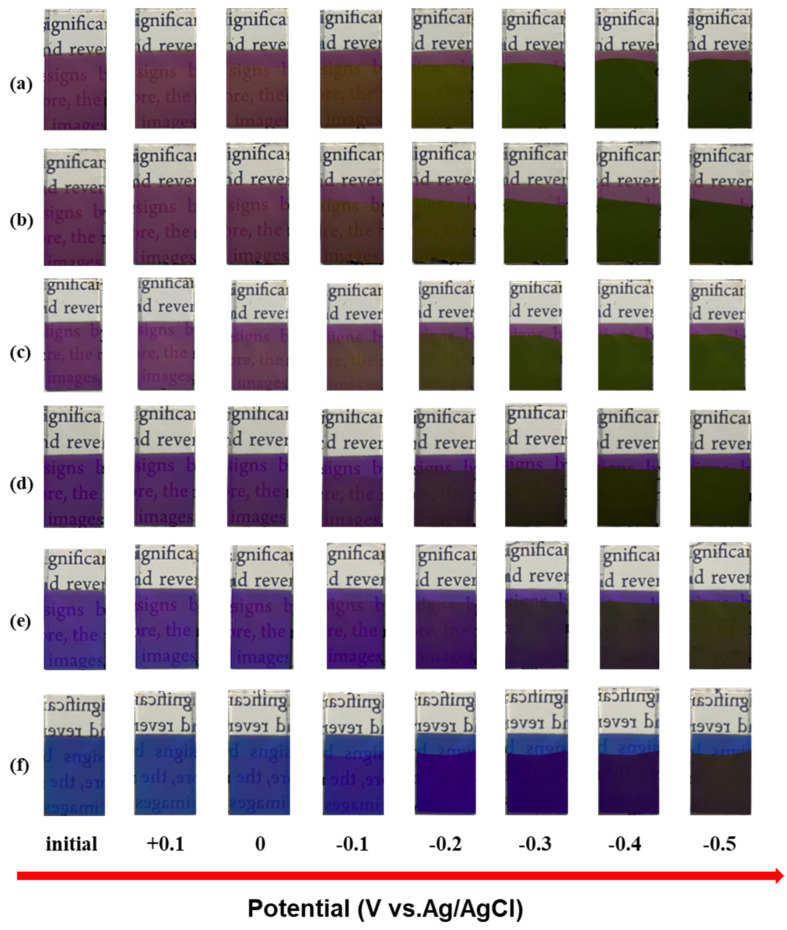
Optical images of different samples with different potentials: (**a**) Film 1, (**b**) Film 2, (**c**) Film 3, (**d**) Film 4, (**e**) Film 5, (**f**) Film 6.

Figure 5 shows the reflection spectra of six thin film electrodes at different potentials. As the voltage gradually decreases to 0, −0.1, −0.2, −0.3, −0.4, and −0.5 V, the reflectance spectrum of the electrode gradually redshifts and the primary reflectance key decreases. Figure 6 shows optical images of six thin film electrodes at different potentials. It can be seen that during the discharge process, the electrode can produce a multicolor state. Accordingly, its reflection spectrum displays a drastic hypsochromic shift in the peak position, achieving very large modulation range (200 nm) compared with the values previously reported for inorganic electrochromic materials. Its color changes are directly related to the large change in the refractive index of the WO_3_ layer [23]. The refractive index of the WO_3_ layer is found to vary with the amount of hydrogen inserted.

**Figure 7:**
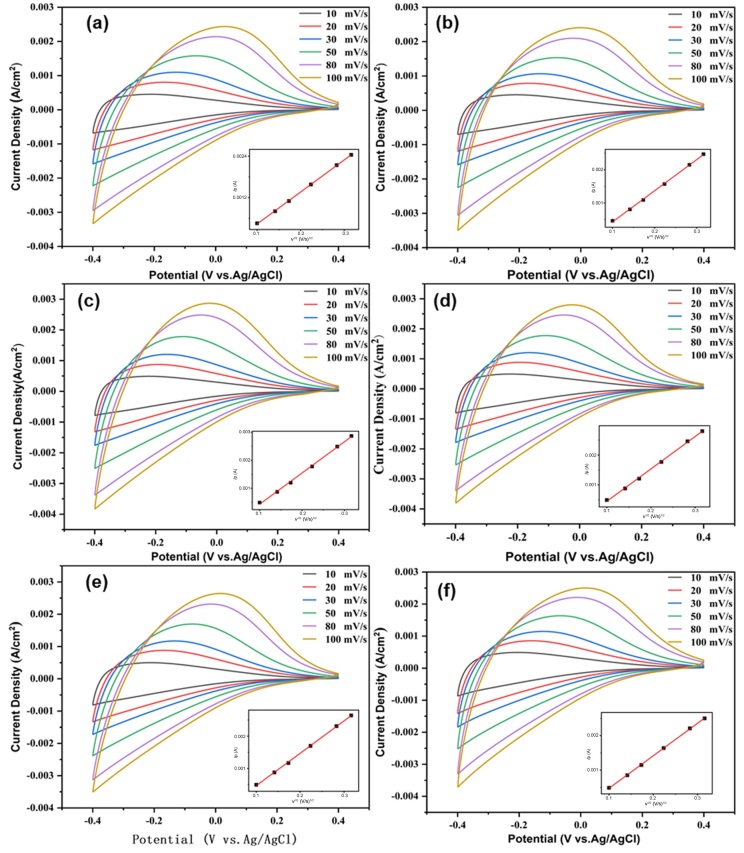
CVs with different scan rates (peak current *I*_p_ as function of square root of scan *v*^1/2^ tested in the inset) for different samples: (**a**) Film 1, (**b**) Film 2, (**c**) Film 3, (**d**) Film 4, (**e**) Film 5, (**f**) Film 6.

Characteristic cyclic voltammogram (CV) curves of multilayer films between −0.4 V and +0.4 V at different scanning rates (10, 20, 30, 50, 80, and 100 mVs^−1^) are shown in Figure 7. All electrode films show broad and featureless CV curves. The CV curves of the films are similar to stoichiometric and nonstoichiometric tungsten oxide analogues [24,25]. All electrode films exhibit distinct oxidation peaks, with no apparent reduction peak. As the potential decreases from +0.4 V to −0.4 V, a significant increase takes place in the cathodic current density associated with the H^+^ ions, and electrons intercalate into the films. Reversing the potential from −0.4 V to +0.4 V, the H^+^ ions and electrons deintercalate out of the films due to the oxidation. These processes lead to the films’ color change. The oxidation peak shifts continuously to higher potentials with the increasing scanning rate. With the increase in the scanning rate, the peak current density gradually increases, indicating that the number of protons and electrons embedded in the film increases, and further indicates that the reactivity of the film is enhanced. The plot of the peak current *I*_p_ as a function of the square root of the scan rate *v*^1/2^ is provided in the insets in Figure 7. *I*_p_ is in direct proportion to *v*^1/2^, confirming the diffusion-controlled behavior.

**Figure 8:**
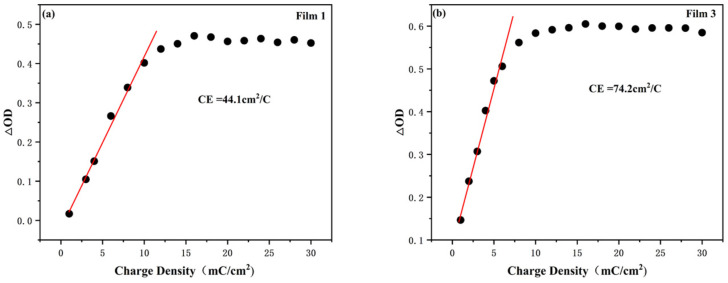
Optical density variation (∆OD) vs. charge density of (**a**) Film 1 and (**b**) Film 3 when monitored at a wavelength of 750 nm.

The optical density (ΔOD) of Film 1 and Film 3 in relation to the charge density at a monitoring wavelength of 750 nm is depicted in Figure 8. Film 1 exhibits a color-rendering efficiency of 44.1 cm^2^ C^−1^, which is consistent with previous reports (CNT/WO_3_ 17.3 cm^2^ C^−1^, WO_3_/W 48.6 cm^2^ C^−1^) and the normal current collector-covered electrochromic electrodes (FTO/WO_3_ 36.1 cm^2^ C^−1^, ITO/WO_3_ 47.1 cm^2^ C^−1^) [23,26]. Film 3 demonstrates a higher efficiency of 74.2 cm^2^ C^−1^. Notably, the W/WO_3_/TiO_2_ electrode displays superior color-rendering efficiency compared to the W/WO_3_ electrode, suggesting that an appropriate thickness of the TiO_2_ layer can effectively enhance its coloring performance.

**Figure 9:**
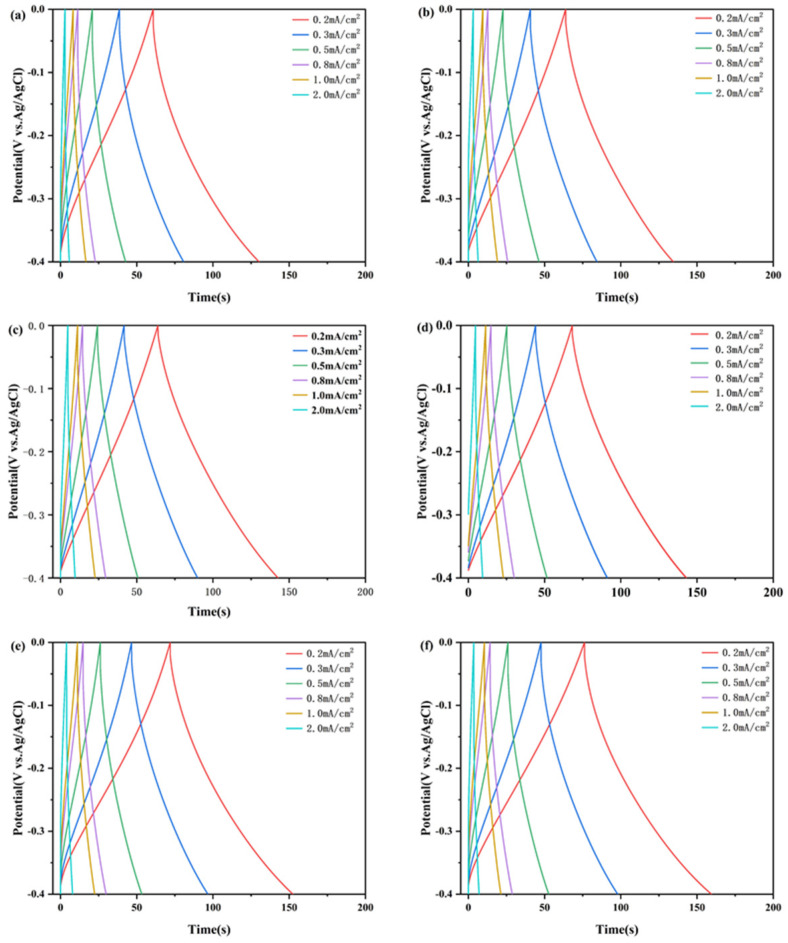
GCD curves of different samples: (**a**) Film 1, (**b**) Film 2, (**c**) Film 3, (**d**) Film 4, (**e**) Film 5, (**f**) Film 6.

**Figure 10:**
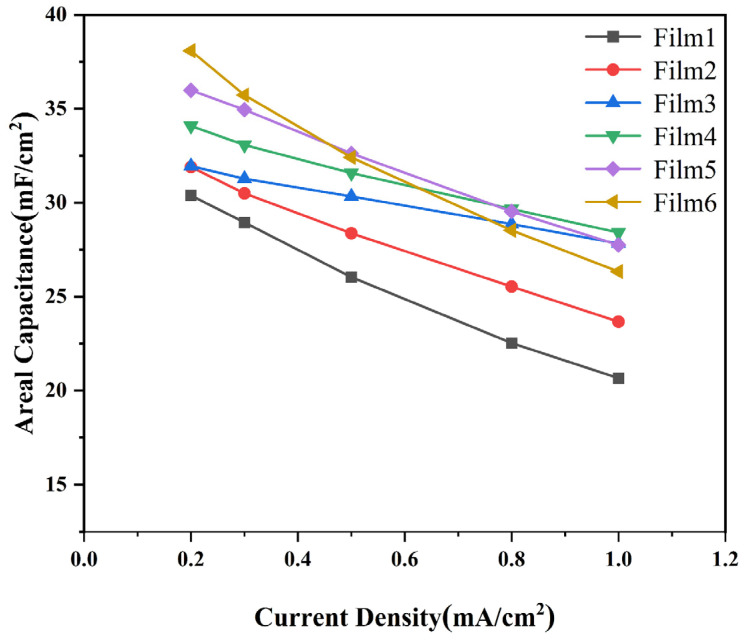
Areal capacitance of samples with different current densities.

Areal capacitances obtained by the galvanostatic charge/discharge (GCD) curves were used to evaluate the capacitance performances of the complex films, as shown in Figure 9. The areal capacitance of the complex films (0.2 mA/cm^2^) were calculated to be 30.4 mF/cm^2^, 31.9 mF/cm^2^, 32.0 mF/cm^2^, 34.1 mF/cm^2^, 35.9 mF/cm^2^, and 38.1 mF/cm^2^. These values indicate that the films’ properties align with those of other tungsten oxide-based supercapacitor electrodes. The areal capacitances of complex films with different current densities were calculated and are shown in Figure 10. Areal capacitance decreases gradually with the increase in discharge current density. The areal capacitance of the W/WO_3_ film retains 68% of the maximum capacitance at a current density of 1 mA/cm^2^, while the areal capacitance of the W/WO_3_/TiO_2_ (10 nm) film retains 87%. This result shows the perfect rate performance of a film electrode with the optimal thickness of the TiO_2_ layer.

**Figure 11:**
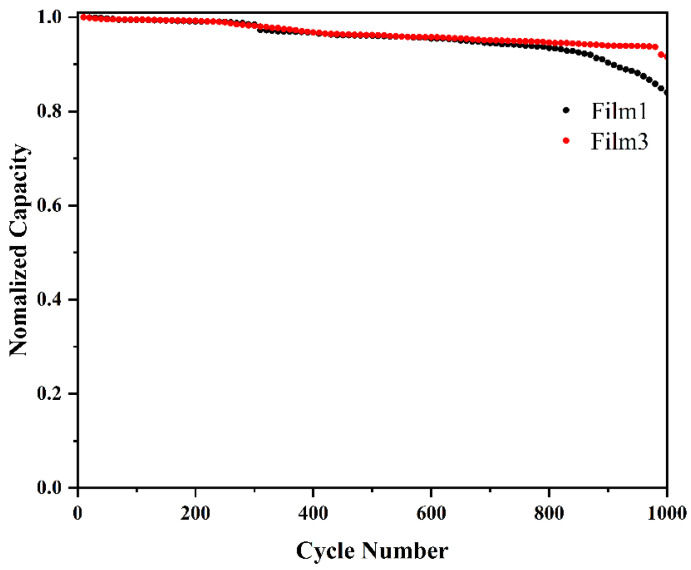
Normalized charge capacity of Film 1 and Film 3 over 1000 cyclic voltammograms (CVs) at a scan rate of 100 mv s^−1^ with the potential range of −0.4 V~+0.4 V.

Continuous cyclic voltammograms (CVs) with a scan rate of 100 mVs^−1^ were used to characterize the stability of Film 1 and Film 3, as shown in Figure 11. Following 1000 cycles, the capacity of Film 1 decreased to 81%, whereas the good stability of Film 3 is demonstrated by a high capacity retention of 91% after 1000 cycles. The surface layer of titanium dioxide can effectively avoid direct contact with electrolytes and protect the WO_3_ film.

## 4. Conclusions

In conclusion, W/WO_3_/TiO_2_ complex films were fabricated. The optical and electrochemical properties of WO_3_ thin films are greatly influenced by the appropriate thickness of surface TiO_2_. The results show that the optimum thickness of the TiO_2_ layer is 10 nm. The as-prepared film displays a high coloration efficiency (CE) of 74.2 cm^2^ C^−1^, a high areal capacitance of 32.0 mF/cm^2^, an excellent rate performance (with the areal capacitance retaining 87% of the maximum capacitance at a current density of 1 mA/cm^2^), and a high cycle life (with a capacity retention 91% after 1000 cycles).

## Data Availability

The original contributions presented in this study are included in the article. Further inquiries can be directed to the corresponding authors.
